# Supplementary material for: Spectrin-beta 2 facilitates the selective accumulation of GABAA receptors at somatodendritic synapses
Source: Commun Biol. 2023 Jan 5;6:11. doi: 10.1038/s42003-022-04381-x (PMC9816108; doi:10.1038/s42003-022-04381-x)
Supplement: Supplementary file 1 — Supplementary Information [file 42003_2022_4381_MOESM1_ESM.pdf]

Supplementary Figure 1.

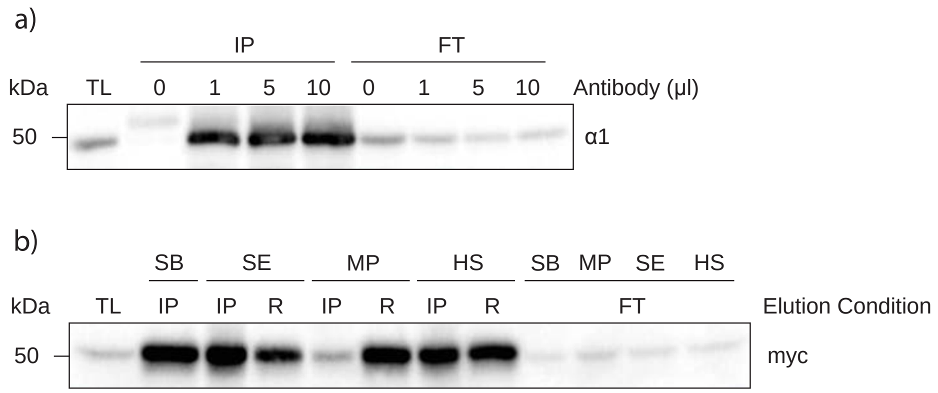


**Immunoprecipitation and elution optimization for a1 and a2.** a) IP efficiency was measured by immunoblot for different bead:antibody ratios. b) Elution efficency was measured by immunoblot following elution (IP) with sample buffer (SB), soft elution buffer (SE), myc peptide (MP), or high salt (HS). Any remaining protein was eluted with sample buffer (R). The amount of protein in the flow through (FT) was also measured.

b)

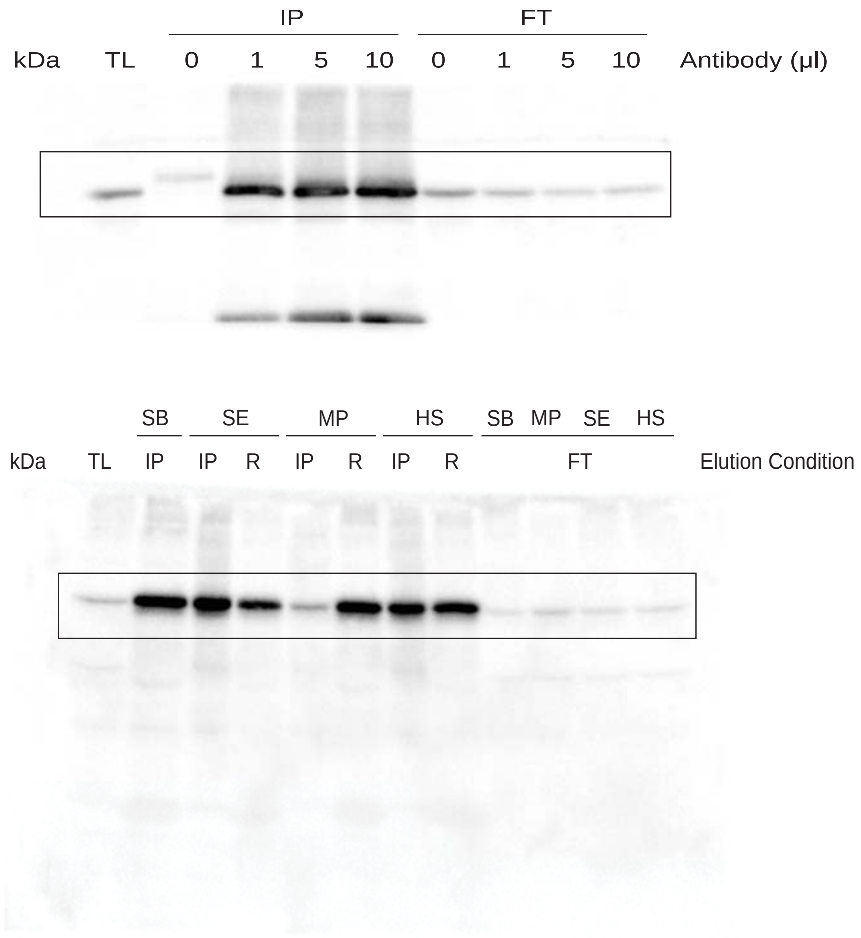


Raw membrane images from the scans shown in Supplementary Figure 1a.

Supplementary Figure 2.

a)

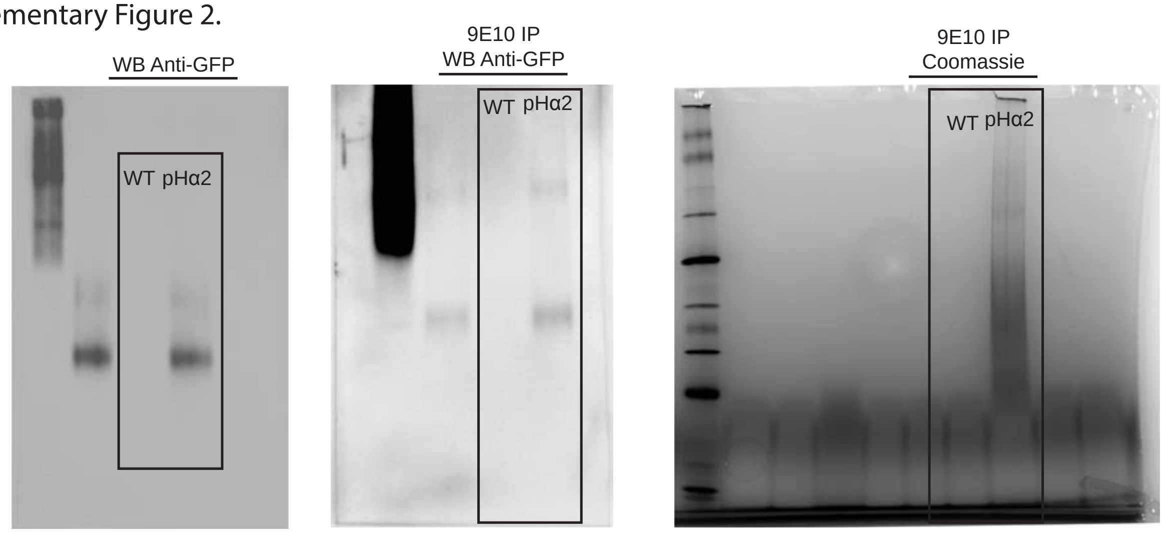


b)

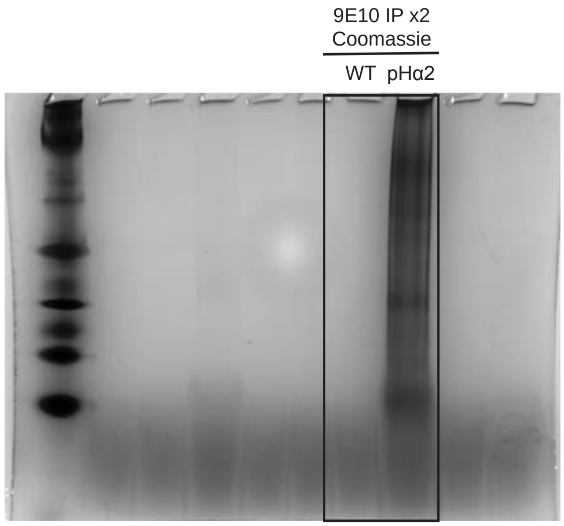


c)

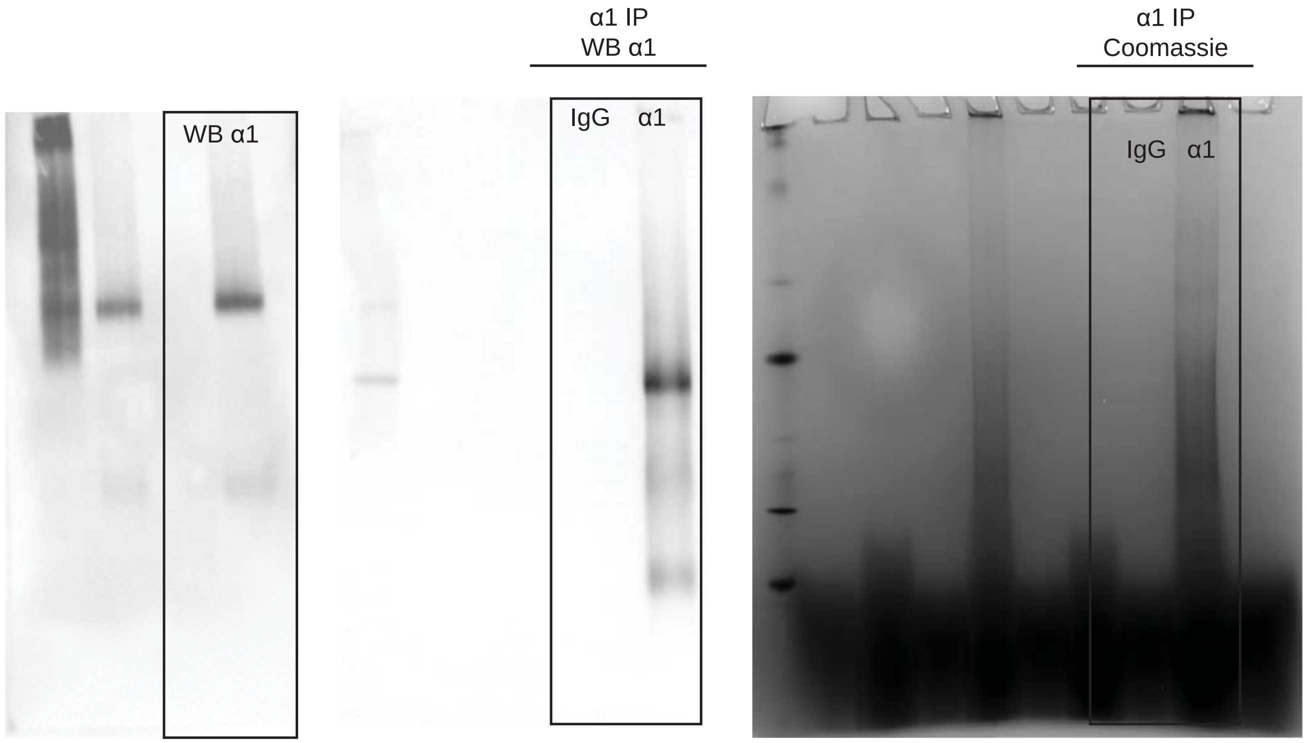


Raw membrane/gel images for the membrane/gel scans shown in Figure 1

Supplemental Figure 3.

a)

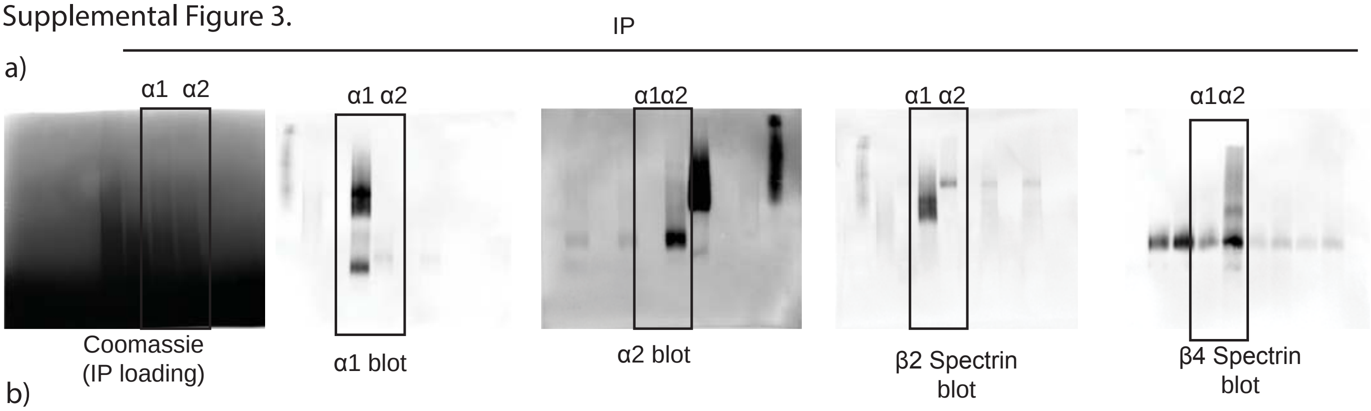


b)

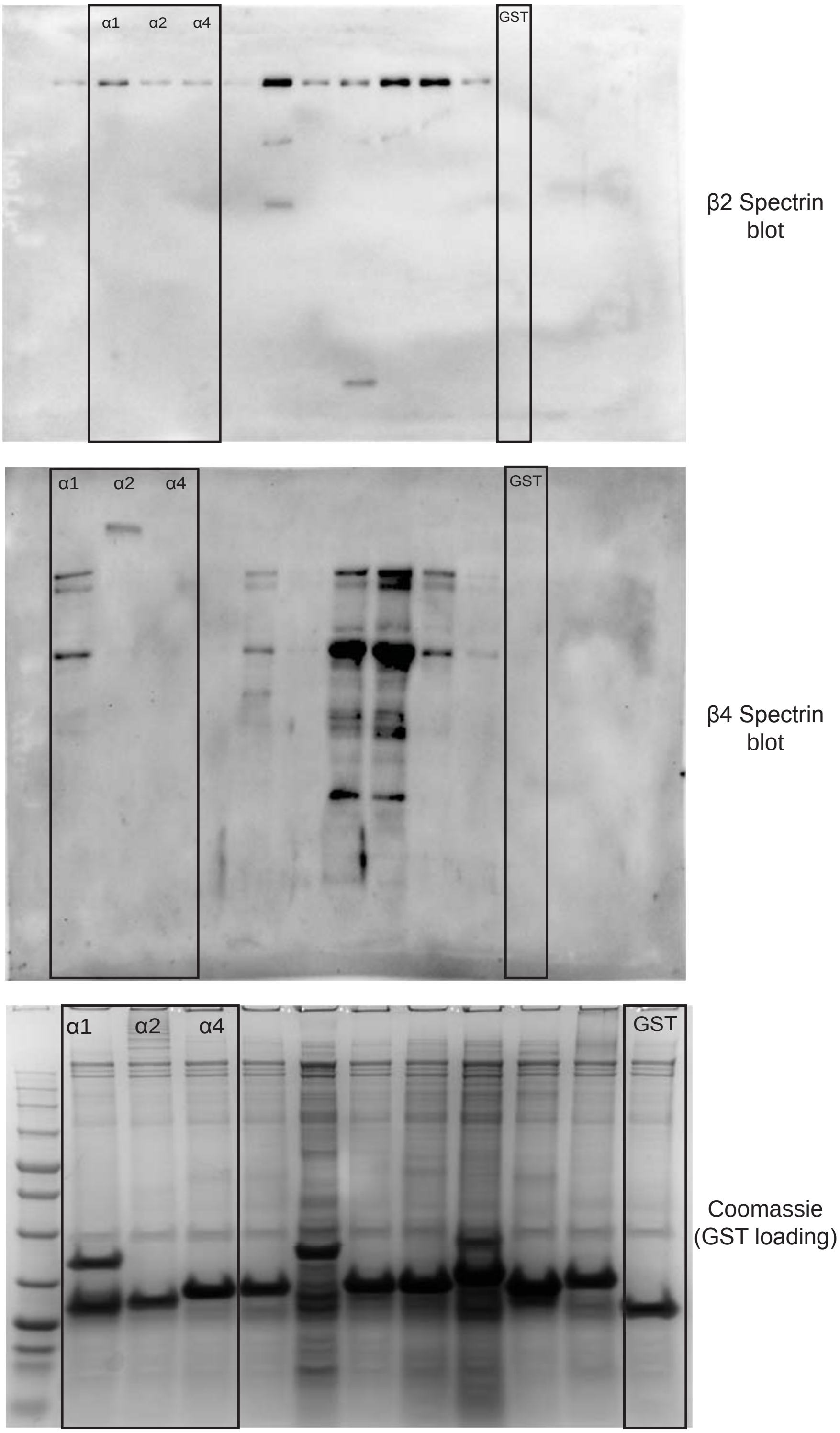


Raw membrane/gel images for the membrane/gel scans shown in Figure 4.

Supplemental Figure 4.

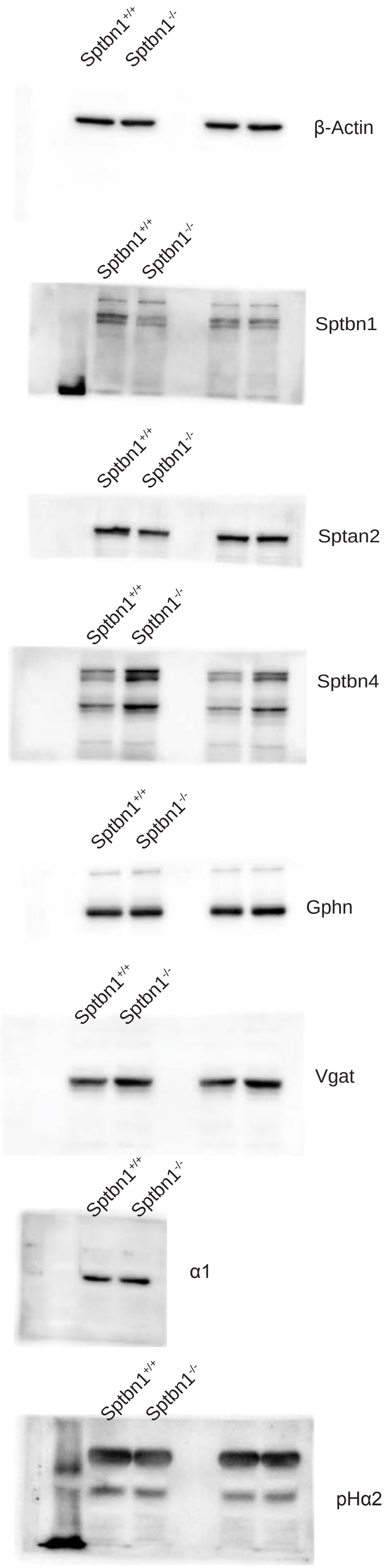


Raw membrane images of the immunoblot scans shown in Figure 5..
