# Supplementary material for: Spectrin-beta 2 facilitates the selective accumulation of GABAA receptors at somatodendritic synapses
Source: Commun Biol. 2023 Jan 5;6:11. doi: 10.1038/s42003-022-04381-x (PMC9816108; doi:10.1038/s42003-022-04381-x)
Supplement: Supplementary file 2 — Description of Additional Supplementary Files [file 42003_2022_4381_MOESM2_ESM.pdf]

File Name: Supplementary Data 1
Description: Data underlying Figure 1b.

File Name: Supplementary Data 2
Description: Data underlying Figure 1d.

File Name: Supplementary Data 3
Description: Data underlying Figure 1f.

File Name: Supplementary Data 4
Description: Data underlying Figure 2b.

File Name: Supplementary Data 5
Description: Table of interactors for Figure 3.

File Name: Supplementary Data 6
Description: Data underlying Figure 4b.

File Name: Supplementary Data 7
Description: Data underlying Figure 5.

File Name: Supplementary Data 8
Description: Data underlying Figure 6.

File Name: Supplementary Data 9
Description: Data underlying Figure 7.
